# Challenging dedifferentiated liposarcoma identified by *MDM2*-amplification, a report of two cases

**DOI:** 10.1186/1472-6890-14-36

**Published:** 2014-07-28

**Authors:** Suvi Lokka, Andreas H Scheel, Sebastian Dango, Katja Schmitz, Rudolf Hesterberg, Josef Rüschoff, Hans-Ulrich Schildhaus

**Affiliations:** 1 Institute of Pathology Nordhessen, Germaniastr. 7, 34119 Kassel, Germany; 2 Department of Pathology, University Medical Centre Göttingen, Robert-Koch-Str. 38, 37077 Göttingen, Germany; 3 Rotes Kreuz Krankenhaus, Department of Surgery, Hansteinstrasse 29, 34121 Kassel, Germany; 4 Department of General, Visceral, and Paediatric Surgery, University Medical Centre Göttingen, Robert-Koch-Str. 38, 37077 Göttingen, Germany

**Keywords:** Dedifferentiated Liposarcoma, Liposarcoma with osteoblastic component, *MDM2*, Fluorescence in situ hybridisation

## Abstract

**Background:**

Liposarcoma is the most frequent soft tissue sarcoma. Well differentiated liposarcoma may progress into dedifferentiated liposarcoma with pleomorphic histology. A minority additionally features myogenic, osteo- or chondrosarcomatous heterologous differentiation. Genomic amplification of the *Mouse double minute 2 homolog (MDM2)* locus is characteristic for well differentiated and dedifferentiated liposarcomas. Detection of *MDM2* amplification may supplement histopathology and aid to distinguish liposarcoma from other soft tissue neoplasia.

**Case presentation:**

Here we present two cases of dedifferentiated liposarcoma with challenging presentation. Case 1 features a myogenic component. As the tumour infiltrated the abdominal muscles and showed immunohistochemical expression of myogenic proteins, rhabdomyosarcoma had to be ruled out. Case 2 has an osteosarcomatous component resembling extraosseous osteosarcoma. The *MDM2* status was determined in both cases and helped making the correct diagnosis. Overexpression of MDM2 and co-overexpression of Cyclin-dependent kinase 4 is demonstrated by immunohistochemistry. The underlying *MDM2* amplification is shown by fluorescence in situ hybridisation. Since low grade osteosarcoma may also harbour *MDM2* amplification it is emphasised that the amplification has to be present in the lipomatous parts of the tumour to distinguish liposarcoma from extraosseous osteosarcoma.

**Conclusions:**

The two cases exemplify challenges in the diagnoses of dedifferentiated liposarcoma. Liposarcoma often has pleomorphic histology and additionally may feature heterologous components that mimic other soft tissue neoplasms. Amplification of *MDM2* is characteristic for well differentiated and dedifferentiated liposarcomas. Determination of the *MDM2* status by in situ hybridisation may assist histopathology and help to rule out differential diagnoses.

## Background

Liposarcoma (LS) is the most common soft tissue sarcoma with about 20% [1]. They are categorized into five subtypes according to WHO classification [2,3] (Table 1). The majority are well differentiated liposarcomas/atypical lipomatous tumours (ALT; 40%). About 10% progress to dedifferentiated liposarcoma [3] (DDLPS). LS are usually located in the deep soft tissue. ALT are most frequently located in the limbs, particularly the thighs while DDLPS are most frequent in the retroperitoneum. LS affect adults, the incidence peaks around 60 years.

**Table 1:** **Benign and malignant lipomatous tumors as listed by the current WHO-definition and *MDM2*-status**

**Entity**	**Dignity**	**ICD-O**	**MDM2 amplified**
Lipoma	Benign	8850/0	no
Lipoblastoma	Benign	8851/0	no
Angiolipoma	Benign	8861/0	no
Myolipoma	Benign	8890/0	no
Chondroid lipoma	Benign	8862/0	no
Spindle cell lipoma	Benign	8854/0	no
Pleomorphic lipoma	Benign	8854/0	no
Hibernoma	Benign	8880/0	no
Well differentiated liposarcoma/atypical lipomatous tumor	Intermediate, locally aggressive	8851/1	yes
Dedifferentiated liposarcoma	Malignant	8858/3	yes
Myxoid liposacroma	Malignant	8852/3	no
Pleomorphic liposarcoma	Malignant	8854/3	no

Histomorphology of DDLPS usually shows remains of ALT with an abrupt shift to dedifferentiated neoplastic tissue. The dedifferentiated tissue is most commonly non lipogenic and pleomorphic, reminiscent of undifferentiated pleomorphic sarcoma (formerly called malignant fibrous histiocytoma). Additionally, heterologous differentiation occurs in about 10% of DDLPS and may present as myogenic, osteo/chondrosarcomatous or angiosarcomatous [4]. Thus DDLPS may mimic a broad spectrum of soft tissue tumours. The clinical prognosis of DDLPS is better than for other high grade sarcoma and is not affected by the presence of heterologous differentiation.

A hallmark of ALT and DDLPS is the genomic amplification of the *MDM2* gene [5]. This can be detected by fluorescence in situ hybridization (FISH) and may facilitate diagnosis [6].

In this report we present two cases of DDLPS with challenging presentations. Both tumours featured heterologous components imitating other soft tissue sarcoma. *MDM2* amplification was detected by FISH and helped to rule out potential differential diagnosis.

## Case presentation

### Case #1, clinical presentation

**Figure 1:**
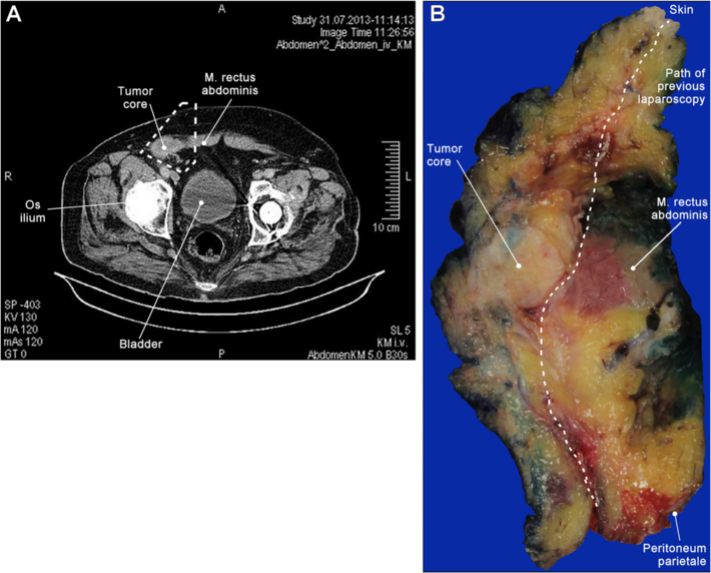
**Clinical presentation of Case #1: Preoperative CT-scan (A) of the tumour in the lower left abdominal wall.** Macroscopic presentation of the surgical specimen **(B)**; central parts of the tumour are well delimited ('core'); the path of the primary laparoscopy is visible. The tumour infiltrated the abdominal skeletal muscles but did not extra into the abdominal cavity.

An 84 year old male obese (BMI 35 kg/m^2^) patient presented with chronic anaemia, localized right abdominal pain and loss of appetite. A CT-scan unmasked a 4.2 × 2.7 × 7 cm hypodense (50HU) solid mass in the painful abdominal region [Figure 1A]. Staging laparoscopy was performed and revealed a whitish tumour in the subcutaneous fatty tissue. The adjacent abdominal skeletal muscles were infiltrated while the peritoneum was not compromised and the abdominal cavity was inconspicuous. Biopsies were taken and routine histology showed a malignant mesenchymal neoplasm. After discussion in an interdisciplinary tumour board wide resection with the aim of complete tumour removal was performed. The tumour region including the path of the laparoscopy, adjacent skin and peritoneum, inguinal canal and ductus deferens were removed en-bloc. The abdominal wall was reconstructed using an intraperitoneal onlay mesh graft technique (IPOM). Histological finding revealed disseminated tumour growth into the cranio-lateral margin (R1). Reoperation yielded a complete tumour removal (R0). The patient recovered and was discharged from hospital 13 days after initial surgery. Soon after the patient was readmitted with ileus due to abdominal adhesions. Laparoscopy was performed and a 35 cm long small intestine segment was removed. Histology did not show any further tumour infiltrates. The patient recovered well and was in good health one year later.

### Histologic and molecular findings

**Figure 2:**
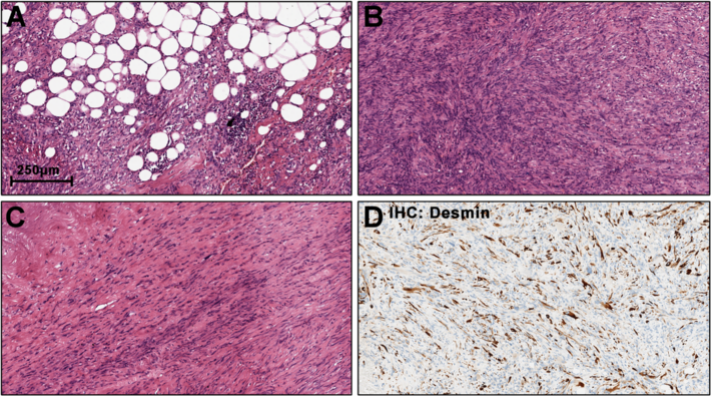
**Histopathology of Case #1: The tumour shows patches of higher differentiated atypical lipomatous tissue (A) but mostly displays only poorly differentiated spindle-shaped cells (B).** Prominent areas with myofibroblastic morphology were noticed **(C)** and immunohistochemistry was positive for Desmin **(D)** and Actin.

On gross examination the 13 × 10.5 × 6 cm specimen contained a 7 × 3.5 × 3.1 cm tumour with whitish/pale yellow cut surfaces [Figure 1B]. The tumour was mostly well delimited with focal areas of diffuse transition into the surrounding tissue. Haematoxylin and eosin (HE) staining showed a neoplasm with high cellularity and mostly spindle-shaped cells arranged in storiform patterns (Figure 2B). Focal transition into more well differentiated atypical adipose tissue were present (2A). Parts of the tumour showed myofibroblastic morphology with parallel, slender cells (2C). Immunohistochemistry (IHC) revealed coexpression of Actin and Desmin (2D) while Caldesmon and Myogenin were negative.

**Figure 3:**
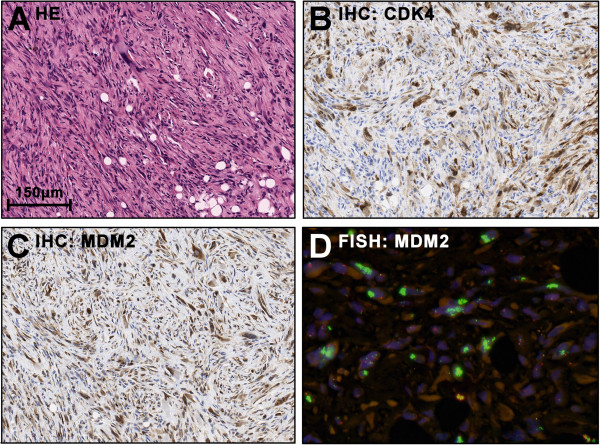
**Molecular hallmarks of Case #1: Immunohistochemistry demonstrates co-overexpression of CDK4 (B) and MDM2 (C) in both the poorly and higher differentiated areas (A: HE-staining of corresponding region).** Fluorescence in situ hybridisation shows strong amplification of the MDM2 locus as underlying genetic alteration (**D**; Green: MDM2 probe, Red: Chromosome 12 reference probe). The features are characteristic of dedifferentiated liposarcoma.

Neoplastic giant cells with nuclear vacuoles were present (Figure 3) and IHC stainings for CDK4 and MDM2 were positive (3B, C). Fluorescence in situ hybridization with a *MDM2*-specific probe (ZytoLight SPEC MDM2/CEN12 dual colour probe; ZytoVision, Bremerhaven, Germany) was supplemented. Groups of highly amplified *MDM2* clusters were detected in all parts of the tumour (3D). Given morphologic and molecular findings, the neoplasm was identified as dedifferentiated liposarcoma with myofibroblastic component (ICD-O: C49.4 M8858/3 G3 (FNCLCC)).

### Case #2, clinical presentation

A 66 year old male patient presented with acute abdomen. The pain was pronounced in the lower right abdomen. Emergency surgery was performed and revealed a ruptured cystic mass of the lower right abdominal wall with connection to the abdominal cavity. En bloc resection was performed. The mass was found to be contained in the soft tissue without connection to the pelvic bones. Given the emergency situation and rupture, excision in toto could not be guaranteed.

### Histologic and molecular findings

Gross examination showed a 20 × 9 × 2.5 cm specimen with yellow-whitish, smooth surface. Cross sections showed soft and fatty tissue. The wall showed areas with solid, partially mineralized tissue with lamellar macroscopic appearance.

**Figure 4:**
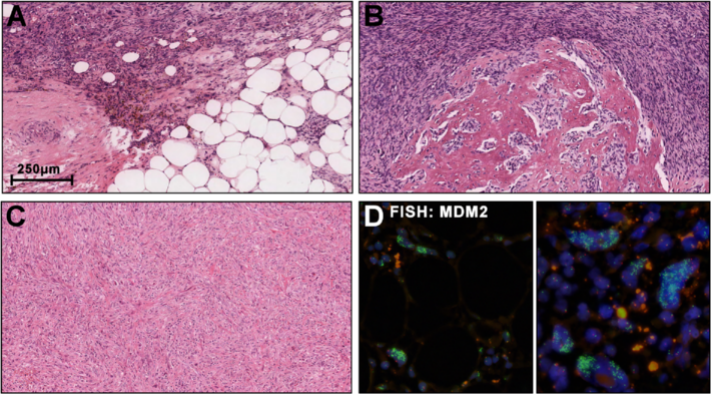
**Histopathology and molecular hallmarks of Case #2: The tumour showed heterogeneous morphology.** Most cells were spindle-shaped but giant cells with segmented nuclei were also present. Focally, transitions into higher differentiated lipomatous tissue were noticed **(A)**. Other areas were of osteoblastic/osteosarcomatous **(B)** and fibrosarcomatous appearance **(C)**. Fluorescence in situ hybridisation of the MDM2 gene revealed highly amplified clusters in the lipomatous (**D**, left), fibrosarcomatous and the osteosarcomatous regions (**D**, right).

HE stainings showed a mesenchymal proliferation with high cellularity and mixed morphology (Figure 4): The cells were mostly of spindle-shaped appearance with high pleomorphy. Giant cells with segmented nuclei were present. Mitoses and few atypical mitoses were observed besides areas with no mitotic activity. Transitions into more differentiated fatty tissue were found (Figure 4A) as well as areas with osteoblastic/osteosarcomatous (4B) and fibrosarcomatous appearance (4C). FISH of the *MDM2* gene revealed highly amplified clusters in the lipomatous, fibrosarcomatous and the osteosarcomatous regions (4D, ZytoLight SPEC MDM2/CEN12 dual colour probe). The tumour was identified as dedifferentiated liposarcoma with heterologous differentiation (ICD-O: C49.4 M8858/3 G2 (FNCLCC)).

### Course of disease

The patient was transferred to a medical centre specialized in abdominal and plastic surgery. Wide excision of the tumour area was performed about one month after initial surgery. Intraoperative two tumours on the small intestine were noticed and excised. Histology confirmed tumour implants of the liposarcoma both of which had been locally excised in sano. The patient recovered from the extended surgical procedures and no signs of recurrent disease were detectable during follow-up CT- and ultrasound-scans one year later.

## Conclusions

Two cases of neoplasm of the lower abdominal wall were resolved as dedifferentiated liposarcomas. Each tumour featured mixed morphology, one with myofibroblastic and one with osteoblastic component. Histopathology was supplemented with detailed immunohistochemistry and in situ hybridization for *MDM2*. Both tumours showed high *MDM2* amplification which is an almost pathognomonic finding given the localization and age of the patients.

ALT and DDLPS usually show a characteristic genomic amplification of 12q13-15 including the proto-oncogenes *MDM2* (12q14.3-15) and *CDK4* (12q14). Amplification may be detected by comparative genome hybridization or by FISH. *CDK4* is often coamplified with *MDM2* and the gene products may be detected by IHC. Ubiquitin ligase MDM2 accelerates protosomal degradation of master tumour suppressor protein p53 while cell cycle associated kinase CDK4 phosphorylates and activates the retinoblastoma gene product (Rb) [7,8]. Physiologically CDK4 is itself activated by D cyclines during G1 progression and inhibited by p16 (INK4a). Conversely p53 point mutations are rarely found in ALT and DDLPS.

The most widely used technique to determine the *MDM2* status is currently dual colour FISH. Two probes specific for 12q14 and for the centromeric region of chromosome 12 are employed to detect amplification or polysomy. Cases are dichotomously classified by the ratio of *MDM2* to centrosome; ≥2.0 are regarded as amplified and <2.0 as non-amplified. At least 50 non overlapping neoplastic cells should be counted [6]. In our own hands application of the threshold is usually not required since ALT and DDLPS invariably show high amplifications with clusters of >10 copies [Figures 2D, 3D].

Sensitivity of the FISH analysis has been reported to be 93.5%-100% in a case-dependent manner [6,9]. It also has a high specificity in distinguishing lipoma from ALT. Benign lipomatous lesions do not harbour *MDM2* amplifications [6,10]. Spindle cell lipoma are reported to frequently have chromosome 12 polysomy [6] while ALT and DDLPS close to always show *MDM2* amplifications. On the other hand, myxoid liposarcomas and most pleomorphic liposarcomas do not harbour *MDM2* amplifications. An exception from the rule of thumb that pleomorphic liposarcomas are *MDM2* negative are cutaneous and subcutanous pleomorphic liposarcoma which may be *MDM2* amplified in very rare cases [11]. A recent study could demonstrate that peripheral undifferentiated sarcomas with *MDM2* amplification correspond to DDLPS, even if a well-differentiated component was not present [12].

Overall, few non-liposarcomatous tumours are known to have increased *MDM2* gene copy numbers. Most notably low grade osteosarcoma arising on the surface of bone may feature 12q14 amplifications [13] while extraosseous osteosarcoma are complex genomic sarcoma without recurrent *MDM2* amplifications.

The two cases exemplify challenges in the diagnosis of DDLPS. Case #1 showed little remaining well differentiated liposarcomatous tissue and featured a myogenic component. Therefore, most important differential diagnosis was rhabdomyosarcoma. Localisation, epidemiology and molecular findings render this possibility unlikely: IHC for Myogenin was negative and the *MDM2* amplification is not observed in tumours of myogenic origin.

Case #2 featured a striking osteosarcomatous component which could also reflect presence of an extraosseous osteosarcoma. Given the localisation close to a fascia ossifying myositis would be benign second differential diagnoses. Transitions into atypical lipomatous tissue were noticed and under the hypotheses of DDLPS hybridisation for *MDM2* was performed. Low grade Osteosarcoma (OS) may also harbour amplified *MDM2*. Thus, the detection of *MDM2*-amplification in the osteosarcomatous parts is not suited to distinguish between low grade OS and DDLPS. However, the amplification in our case was present in all parts of the tumour, including the atypical adipocytes. Thus the malignant character of the atypical adipose tissue, i.e. the liposarcomatous tissue was confirmed. Also, the tumour did not have contact to bone and extraosseous OS do not harbour *MDM2* amplifications. Thus, the combination of patient age, localisation, morphology and *MDM2* status strongly argues for the osteosarcomatous tissue to be heterologous component of a DDLPS.

The current standard therapy of DDLPS is wide excision. No consensus exists about the minimal length of the resection margins and the widest resection possible should be archived [14]. This is particularly challenging in the retroperitoneum which may explain the high rates of local recurrences of DDLPS (20-100% depending on the respective study [3]). DDLPS may spread to distant sites in 15-20% of cases while ALT do not metastasise [2]. Case #2 exemplifies that scattered DDLPS tissue may easily spawn tumour implants. Both patients were in good health one year after treatment and showed no signs of recurrent disease. However, given the slow-growing behaviour of DDLPS careful follow-up investigations are necessary.

The recurrent *MDM2* amplification might be a key to targeted therapy of DDLPS: Experimental MDM2-inhibitors have been successfully employed in vitro to reactivate p53 and mediate apoptosis [15]. While the first generation of MDM2 inhibitors proved to be clinically intolerable several newly developed substances are currently undergoing phase I trials. Should they translate into approved drugs the *MDM2* status might become a predictive biomarker.

### Keypoints

Liposarcoma is the most frequent soft tissue neoplasm. About 40% are well differentiated liposarcoma which may progress into dedifferentiated liposarcoma. The clinical prognosis is better compared to other high grade soft tissue sarcoma.

Dedifferentiated liposarcoma has pleomorphic histomorphology. The diagnosis is facilitated by the demonstration of remaining well differentiated liposarcomatous tissue.

A minority of cases may present a heterologous differentiation of myogenic, osteo-/chondrosarcomatous or angiosarcomatous appearance. These have to be distinguished from sarcoma of other origin.

Detection of MDM2 and CDK4 amplification and coexpression by FISH and IHC has a high specificity and may support classification of challenging cases.

## Consent

Written informed consent was obtained from the patients for publication of this case report and any accompanying images. A copy of the written consent is available for review by the Editor of this journal.

## Abbreviations

CDK4: Cyclin dependent kinase 4; FISH: Fluorescence in situ hybridization; FNCLCC: French Fédération Nationale des Centres de Lutte Contre le Cancer grading system; HE: Haematoxylin and eosin; IHC: Immunohistochemistry; MDM2: Murine double-minute 2; OS: Osteosarcoma.

## Competing interests

The authors declare no conflicts of interest. The authors received no funding or financial compensation for the preparation of the manuscript.

## Authors’ contributions

The patient of case #1 was treated by RH and SD. The surgical specimens of both cases were examined by SL and AS. Histopathology was performed by SL and AS under guidance of JR. HUS was contacted for second opinion and for *MDM2*-FISH. FISH was performed by KS and HUS. The manuscript was drafted by SL, AS and SD under guidance of HUS and with contribution of all co-authors. The final manuscript was read and agreed upon by all authors.

## Pre-publication history

The pre-publication history for this paper can be accessed here:

http://www.biomedcentral.com/1472-6890/14/36/prepub
